# Nano-Polymers as Cas9 Inhibitors

**DOI:** 10.3390/polym17030417

**Published:** 2025-02-05

**Authors:** Oksana Chepurna, Avradip Chatterjee, Yuanqing Li, Hong Ding, Ramachandran Murali, Keith L. Black, Tao Sun

**Affiliations:** 1 Department of Neurosurgery, Cedars-Sinai Medical Center, Los Angeles, CA 90048, USA; 2 Department of Biomedical Sciences, Research Division of Immunology, Cedars-Sinai Medical Center, Los Angeles, CA 90048, USA; 3 Shanghai Institute of Material Medica, Shanghai Institutes of Biological Sciences, Chinese Academy of Sciences, Shanghai 200231, China; 4 School of Traditional Chinese Materia Medica, Shenyang Pharmaceutical University, Shenyang 110016, China

**Keywords:** CRISPR/Cas9, nano-polymer, inhibitor, polymalic acid, polyglutamic acid, polyaspartic acid, trileucine

## Abstract

Despite wide applications of CRISPR/Cas9 technology, effective approaches for CRISPR delivery with functional control are limited. In an attempt to develop a nanoscale CRSIPR/Cas9 delivery platform, we discovered that several biocompatible polymers, including polymalic acid (PMLA), polyglutamic acid (PGA), and polyaspartic acid (PLD), when conjugated with a trileucine (LLL) moiety, can effectively inhibit Cas9 nuclease function. The Cas9 inhibition by those polymers is dose-dependent, with varying efficiency to achieve 100% inhibition. Further biophysical studies revealed that PMLA-LLL directly binds the Cas9 protein, resulting in a substantial decrease in Cas9/sgRNA binding affinity. Transmission electron microscopy and molecular docking were performed to provide a possible binding mechanism for PMLA-LLL to interact with Cas9. This work identified a new class of Cas9 inhibitor in nano-polymer form. These biodegradable polymers may serve as novel Cas9 delivery vehicles with a potential to enhance the precision of Cas9-mediated gene editing.

## 1. Introduction

Since its conceptualization, CRISPR/Cas9-mediated gene editing has been rapidly changing the paradigm of life science in the past decade owing to its ease of use and programmable gene editing capability [1,2,3]. However, several technical hurdles, such as difficulty in delivery and potential unwanted (e.g., off-site-on-target and off-target) editing, remain to be overcome [4,5].

We sought to develop an efficient CRISPR delivery system by using nano-polymer technology. A naturally occurring polymer, poly-(β-L-malic acid) (PMLA), derived from an acellular slime mold was employed mainly due to its biocompatibility [6,7]. Owing to its biodegradable property and a decent capacity for covalent conjugation through various -COOH groups, PMLA polymers have been explored as nanodrug carriers in various areas [7,8,9,10]. We have previously engineered the PMLA nano-polymer to carry different payloads, including anti-sense nucleotides, antibodies, and proteins, to treat brain cancer and Alzheimer’s disease in multiple preclinical models [9,10,11,12]. Generally, these therapeutic agents are covalently conjugated with the PMLA backbone through a disulfide (S-S) bond that enables the cargo release into the cytoplasm due to the S-S bond breakage in response to the highly reducing subcellular environment. In addition to the therapeutics, various targeting mechanisms are also conjugated to the PMLA polymer backbone to allow cell- or tissue-specific drug delivery. After internalization by the target cells, to avoid degradation of payloads in the late endosome and lysosome, a short peptide consisting of three leucine resides is also conjugated to the PMLA backbone to allow endosomal escape by breaking the endosome membrane in a pH-dependent fashion [13]. Surprisingly, when we mixed the LLL-conjugated PMLA polymer (PMLA-LLL) with the Cas9 in a DNA cleavage assay, we found the PMLA-LLL was able to completely block the endonuclease activity of the Cas9 protein. Intrigued by this finding, we wanted to determine whether there is a common structural basis of nano-polymers that may affect Cas9 nuclease function and investigated four different nano-polymers that have been widely used. After conjugation with the LLL moiety, we found that two out of four different polymers can also block Cas9 function with varying efficiency.

Multiple Cas inhibitors have been identified, and these Cas inhibitors exhibit a wide variety of inhibitory mechanisms. Generally, Cas inhibitors include naturally occurring proteins, small molecules, and synthetic peptides or nucleotides by rational design [14,15]. For instance, the most abundant naturally occurring Cas inhibitors are anti-CRISPR (Acr) proteins [16,17,18]. Currently, there are more than 20 different families of Acr proteins found in bacteriophages [19,20]. Meanwhile, significant efforts have been made to apply CRISPR/Cas inhibitors to modulate gene editing with precision, and these tools have shown great promise in the temporal control of Cas9 function.

## 2. Materials and Methods

### 2.1. Plasmid DNA, Cas9 and sgRNA

Recombinant SpCas9 protein was purchased from Intact Genomics (St Louis, MO, USA). DNA plasmid pGEM-3Zf(+) was purchased from Promega (Madison, WI, USA). SpCas9-mediated DNA cleavage assays were performed in Cas9 reaction buffer 3.1 recommended by New England Biolabs (NEB), which we refer to as NEB buffer 3.1. The plasmid (3197 bp) was linearized by Pci-I restriction enzyme (New England Biolabs, Ipswich, MA, USA) and was used as the Cas9 DNA cleavage substrate. sgRNA (TCGTAGTTATCTACACGACG; PAM GGG) targeting the ampicillin resistance gene in the pGEM vector was designed by using Broad Institute Cas9 sgRNA design tool (https://portals.broadinstitute.org/gpp/public/analysis-tools/sgrna-design; accessed on 21 December 2020) and was synthesized by Synthego (Redwood City, CA, USA).

### 2.2. Chemical Reagents

Naturally occurring polymalic acid (PMLA) polymer backbone was produced using the culture broth of Physarum polycephalum as previously described [21]. All other polymers used in this study were purchased from commercial sources. Poly-L-glutamic acid (PGA) was purchased from Alamanda Polymers (Huntsville, Al, USA); Poly-L-aspartic acid (PLD), polyacrylic acid (PAA), and Hyaluronic acid (HA) were purchased from ThermoFisher Scientific (Canoga Park, CA, USA). Trileucine (LLL) was ordered from Bachem (Torrance, CA, USA). N,N′-Dicyclohexylcarbodiimide (DCC), N-hydroxysuccinimide (NHS), trifluoroacetic acid (TFA), cysteamine (or 2-mercaptoethylamine, MEA), dithiothreitol (DTT), deuterated acetone and dimethylsulfoxide (DMSO), dimethylformamide (DMF), triethylamine (NEt3), and phosphotungstic acid (PTA) were obtained from Sigma-Aldrich (St. Louis, MO, USA). PD-10 columns were purchased from GE Healthcare (Chicago, IL, USA).

### 2.3. Conjugation of Nano-Polymers

The LLL-conjugated nano-polymers were synthesized as reported previously [7]. Briefly, 40% of malyl residues carried LLL and 10% mercapto ethylamine (MEA) groups that were attached at pendant carboxylates of the polymer. Conjugates were made using a pre-conjugate synthesized by the activation of PMLA pendant carboxylates applying the DCC/NHS method to attach LLL and 2-mercapto ethylamine in a follow-up reaction. N-ethylmaleimide was used to cap the free SH groups. The reaction was monitored using TLC and ninhydrin reaction. The product was purified over a PD-10 column and characterized by SEC-HPLC (7.2 min retention time, 220 nm).

### 2.4. Cas9 DNA Cleavage Assay

A 30 µL total reaction was used to test the Cas9 nuclease activity in DNA cleavage assay, which consists of 3 µL of 10× NEB buffer 3.1, 1 µL of Cas9 nuclease (1 µM), 3 ul of sgRNA (300 nM), 3 µL of linearized pGEM vector DNA (30 nM), and various volumes of nano-polymer or water. The reaction (except substrate DNA) was first mixed at room temperature for 10 min before adding linear DNA substrate. DNA cleavage reaction was incubated at 37 °C for 1 h followed by 1 µL of proteinase K treatment (10 min, room temperature). The DNA cleavage product was visualized by 1% agarose gel electrophoresis. All experiments were repeated at least 3 times and representative images are shown in the figures.

### 2.5. HPLC Assay

The analysis was performed with Agilent Technologies 1260 Infinity II with Software Agilent 1260 Infinity online (Version D.07.41). The SEC-HPLC column was Polysep 4000, at 1 mL/min flow rate and PBS (pH 7.4).

### 2.6. Microscale Thermophoresis (MST) Assays

The 6xHistidine-tag at the N-terminal of Cas9 was fluorescently labelled using NTA labelling kit (NanoTemper, Watertown, MA, USA) using manufacturer’s protocol. All binding assays were performed in buffer containing 20 mM HEPES 7.5, 150 mM NaCl, 5 mM MgCl2, 0.05% Tween20. sgRNA, PMLA, or PMLA-LLL were titrated from 15.25 picomolar (pM) to 1 μM against 67.5 nM Cas9. For the competition assays, 67.5 nM of Cas9 and either PMLA or PMLA-LLL were mixed (equimolar ratio) and incubated at 4 °C for 15 min before titrating sgRNA from 15.25 pM to 1 μM. MST measurements were carried out in Monolith NT.115 and the data were processed in MO (NanoTemper Technologies, Munich, Germany). In MST assay, the dissociation constant (KD) between a fluorescently labeled target (here Cas9), present at a fixed concentration, and a ligand (here sgRNA, PMLA, or PMLA-LLL), which is titrated over a range of concentrations, is measured by detecting the change in fluorescence (indicated as ‘ΔFnorm’ in the graphs) of the analyte as a function of the ligand concentration.

### 2.7. Transmission Electron Microscopy (TEM) Assay

To be imaged with TEM, 10  µL of polymer suspension was dropped onto the carbon support films stabilized with formvar. In order to visualize polymers, they were negatively stained with 1% water solution of phosphotungstic acid prior to dropping onto the support films. The TEM imaging of polymers was performed using FEI Tecnai TF20 transmission electron microscope. For PGA-LLL + Cas9 imaging 4.3 µL of Cas9 with 1 µM concentration with 25 µL of PGA-LLL from 3 µM stock were mixed with molar ratio 18:1.

### 2.8. Molecular Docking

All molecular docking simulations were generated by Maestro v10.2. (Schrodinger, LLC., New York, NY, USA). Firstly, three optimized conformations of each monomer were generated by ligand preparation modules with force field of OPLS3, which were applied as ligands for docking. Then, structures of SpCas9 (PDB: 5VW1) were optimized from Protein Preparation Wizard module, including procedures of adding hydrogens, adding missing sidechains, assigning bond orders, and restraining minimization. The docking processes were conducted by Induced-Fit Docking module with force field of OPLS_2005 and the box center was set to be the center of AcrIIA4 [22]. All generated poses were sorted by the IFD_score and top scored poses were selected for further analysis.

## 3. Results

### 3.1. PMLA-Trileucine (LLL) Conjugate Binds to Cas9 Protein

**Figure 1:**
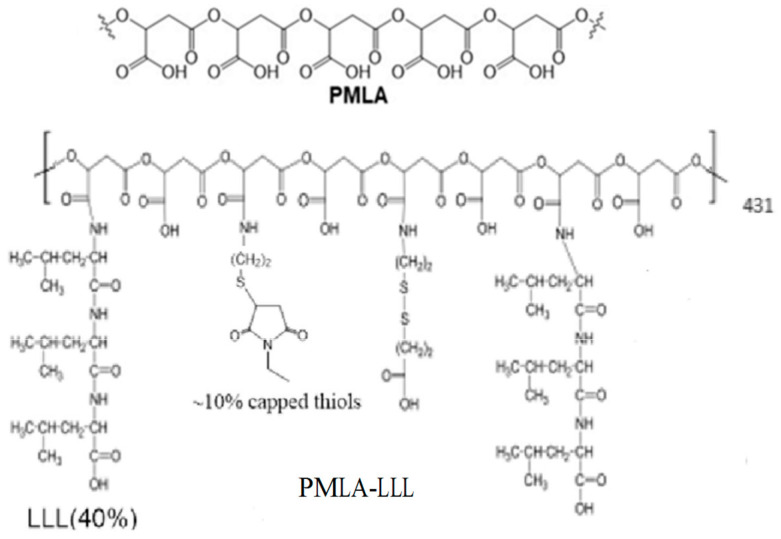
Structure of naturally occurred PMLA polymer and PMLA-LLL conjugates.

PMLA is a naturally occurring linear polymer with an abundance of -COOH groups that allows covalent conjugation of multi-functional modalities, such as tumor targeting mechanisms, therapeutics, and BBB-crossing moieties [23,24,25]. Among these conjugated functional modalities is a trileucine (LLL) peptide that we have used to facilitate endosomal escape after the internalization of nanoconjugates. The chemical structures of PMLA-polymer and PMLA-LLL are shown in Figure 1.

**Figure 2:**
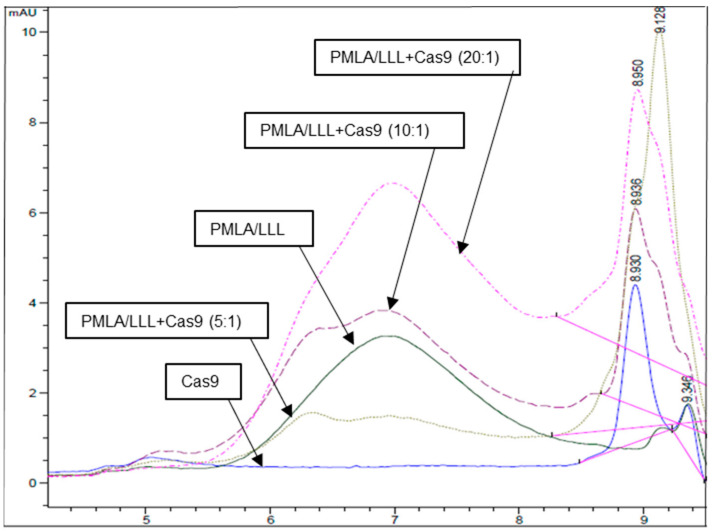
Chromatograms from HPLC analysis demonstrating binding interaction between PMLA-LLL and Cas9 protein at different molar ratios (5:1, 10:1, and 20:1). Free Cas9 and PMLA-LLL alone are represented by distinct peaks, while dose-dependent binding is evidenced by increasing intensity of PMLA-LLL/Cas9 complex peak at higher molar ratios.

To examine the potential interaction of PMLA-LLL with the Cas9 protein, we performed HPLC assays on various formulations, including free Cas9, PMLA-LLL alone, and mixtures of PMLA-LLL with Cas9 at molar ratios of 5:1, 10:1, and 20:1 (Figure 2). The chromatographic profiles reveal a clear interaction between PMLA-LLL and Cas9, with a dose-dependent pattern. Free Cas9 exhibits a distinct peak at approximately ~9 min, which represents its unbound state in the absence of PMLA-LLL. In contrast, PMLA-LLL alone shows a unique peak at a separate retention time, indicating its individual chromatographic behavior. Once mixed with PMLA-LLL at a 5:1 molar ratio, the Cas9 peak decreased in intensity, accompanied by the appearance of a new peak corresponding to the PMLA-LLL/Cas9 complex. Increasing the molar ratio to 10:1 and 20:1 resulted in further reduction of the free Cas9 peak and a simultaneous increase in the complex peak, demonstrating stronger interaction at higher PMLA-LLL concentrations. These findings indicate that PMLA-LLL binds Cas9 in a dose-dependent manner, with optimal binding observed at a 20:1 molar ratio. This property underscores the potential of PMLA-LLL as a nano-polymer carrier for the efficient delivery of CRISPR/Cas9 systems.

In addition, when we mixed the Cas9 protein with PMLA or PMLA-LLL, in the presence or absence of sgRNA, we found that while adding PMLA to either Cas9 protein or sgRNA had a minimal impact on the measured zeta potential [26], a measure of the electric potential at the boundary between a solid particle and its surrounding liquid environment and a low zeta potential means particles are more likely to attract and agglomerate. In contrast, adding PMLA-LLL dramatically decreased the zeta potential, suggesting a much stronger interaction between PMLA-LLL and the Cas9 protein (Table 1).

Next, we performed microscale thermophoresis (MST) to measure the potential binding affinity between the nano-polymers with the Cas9 protein (Figure S1). In this experiment, we first compared the binding affinity of Cas9 to a synthetic sgRNA, a PMLA polymer backbone, and a PMLA-LLL conjugate. Our results showed that Cas9 has the strongest binding to the sgRNA with a dissociation constant (KD) of 1.24 nM, followed by the interaction between Cas9 and PMLA-LLL with a KD value of 3.6 nM (Table 2). The PMLA backbone alone also exhibited some binding affinity to Cas9 with a 10-fold higher KD (36 nM). Interestingly, when we pre-incubated the Cas9 protein with PMLA-LLL or PMLA, we found that the Cas9-sgRNA binding was dramatically decreased, especially with PMLA-LLL preincubation that led to a 425-fold decrease in the Cas9-sgRNA binding affinity (Table 2).

**Figure 3:**
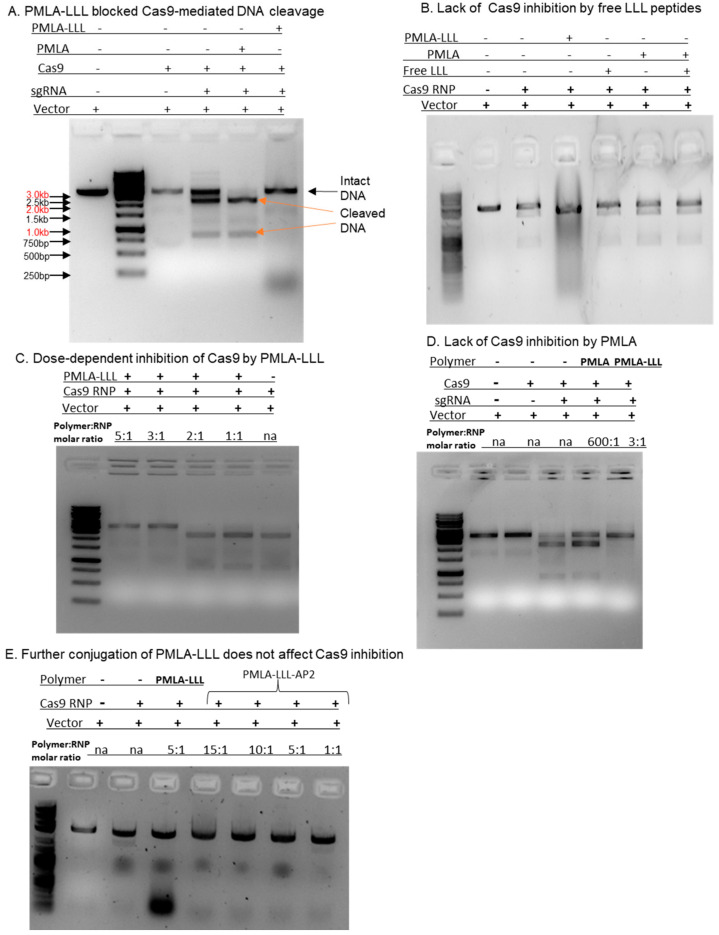
Effects of Cas9 inhibition by PMLA-LLL and PMLA.

Upon confirmation of the binding affinity between the PMLA-LLL polymer and Cas9 protein, we asked whether this interaction may have any impact on Cas9 nuclease function. To answer this question, we used a pGEM vector and designed an sgRNA (TCGTAGTTATCTACACGACG) targeting the ampicillin resistance gene in this plasmid. The pGEM plasmid was first linearized by the Pci-I restriction enzyme, resulting in 3.2 kb linear DNA. After cleavage by Cas9/sgRNA ribonucleoprotein (RNP), this vector DNA would produce two fragments of 2.3 kb and ~900 bp in length. When the Cas9/sgRNA was pre-incubated with the PMLA-LLL polymer, we found that the DNA cleavage was completely blocked, while PMLA incubation did not affect the DNA cleavage by Cas9/sgRNA RNP (Figure 3A).

Next, we asked whether we could achieve the same Cas9 inhibition by incubating the Cas9 protein with the pre-mixed PMLA polymer and free LLL peptide, since we found that the PMLA backbone failed to inhibit Cas9 function (Figure 3A). Our DNA digestion results clearly demonstrated that PMLA plus free LLL peptide had no effect on Cas9 activity (Figure 3B). Furthermore, when we incubated Cas9 with a concentration series of PMLA-LLL with a polymer/Cas9–sgRNA molar ratio of 5:1, 3:1, 2: 1, and 1:1 (Figure 3C), we observed a dose-dependent pattern of Cas9 inhibition by PMLA-LLL, with a minimal 3:1 polymer/Cas9 molar ratio to completely block Cas9 nuclease activity. Lastly, although PMLA showed a moderate binding affinity to Cas9 via the MST assay (Table 2), we did not detect any functional inhibition of Cas9 nuclease activity by PMLA even at an extremely high polymer concentration (PMLA/Cas9 = 600:1) (Figure 3D), suggesting that the naked PMLA backbone lacks a structural basis to block Cas9 function.

Tumor-targeting mechanisms are essential to allow nano-polymers to selectively bind to the tumor cell surface. This tumor-specific binding can be achieved by conjugating an antibody or a peptide with a selective binding affinity to cancer cells, such as an AP-2 (angiopep-2) peptide [7,27]. The next question we asked was whether the addition of a tumor-specific targeting moiety could affect Cas9 inhibition by PMLA-LLL. To answer this question, we modified the PMLA-LLL polymer by further conjugating an AP-2 peptide to generate PMLA-LLL-AP2. AP-2 is a 19-amino-acid synthetic peptide (TFFYGGSRGKRNNFKTEEY) that can specifically interact with the LRP-1 receptor to facilitate blood–brain barrier crossing [28]. In the DNA digestion assays, our data showed that further conjugation of AP-2 to the PMLA-LLL does not affect Cas9 inhibition. As shown in Figure 3E, compared to PMLA-LLL, the PMLA-LLL-AP2 exhibited a similar dose-dependent inhibition of Cas9 with a minimal polymer/RNP molar ratio of 5:1 to completely block Cas9 function.

- PMLA-LLL can effectively block Cas9-mediated DNA cleavage. A linearized pGEM DNA vector (~3.2 kb) was used as the DNA substrate and a sgRNA targeting the ampicillin resistance gene sequence in the vector was used to direct the Cas9-mediated DNA cut. Only when the Cas9/sgRNA RNP was present in the digestion reaction was the DNA vector cut, resulting in two fragments (~2.3 kb and 900 bp). However, when pre-incubated with PMLA-LLL (with a molar ratio of polymer/RNP = 50:1), the DNA digestion was completely blocked.
- A free LLL peptide alone or in combination with a naked PMLA backbone cannot inhibit Cas9 activity.
- The dose-dependence of PMLA-LLL in Cas9 inhibition: a dosage titration of PMLA-LLL incubated with Cas9 demonstrated a minimal ratio of polymer/RNP = 3:1 to inhibit Cas9 nuclease function by 100%. (NA: not applicable).
- Naked PMLA polymer does not inhibit Cas9 function even at an extremely high concentration: polymer:RNP = 600:1.
- Further conjugation of the PMLA-LLL polymer with a tumor-targeting peptide (AP-2) does not affect its Cas9 inhibitory effect.

### 3.2. LLL-Polymer Structure as a Common Mechanism for Cas9 Inhibition

**Figure 4:**
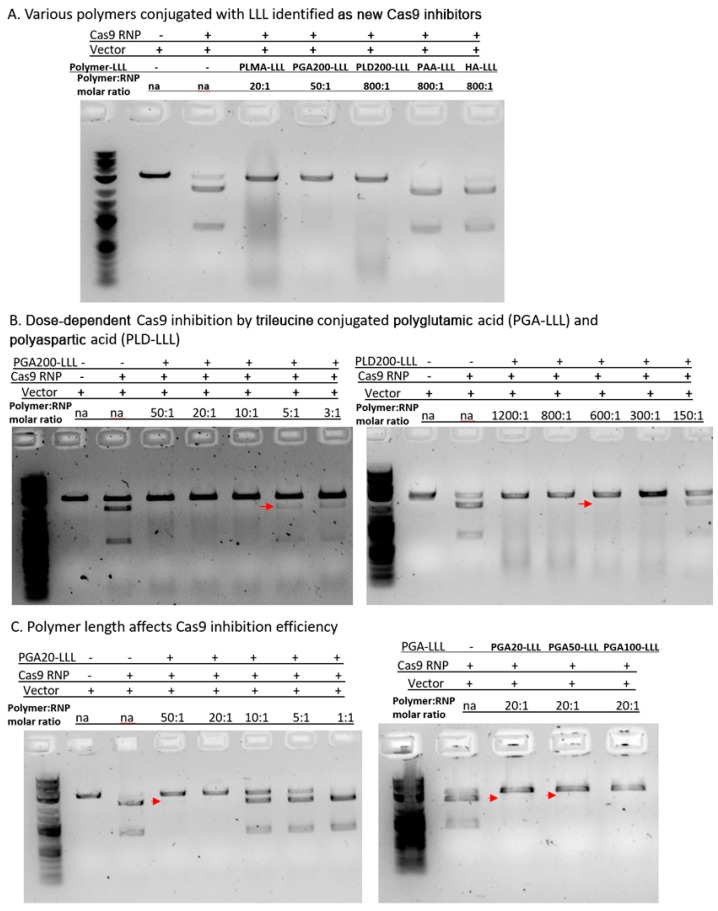
Polymer-LLL conjugates may function as a common mechanism for Cas9 inhibition.

The discovery of PMLA-LLL as a new Cas9 inhibitor prompted us to ask a more fundamental question, which was whether the LLL conjugated polymers may serve as a common Cas9 inhibitory mechanism. To address this question, we conjugated the LLL to several other polymers, including poly-L-glutamic acid 200 (PGA200; number stands for the number of units in the structure), poly-L-aspartic acid 200 (PLD200), polyacrylic acid (PAA), and hyaluronic acid (HA) (Table 3). We screened these LLL-conjugated polymers for their potential inhibitory effects on Cas9 activity, and DNA cleavage assays demonstrated that, besides PMLA-LLL, both PGA200-LLL and PLD200-LLL exhibited a complete inhibition of Cas9 nuclease function, while PAA-LLL and HA-LLL showed no effect (Figure 4A). Meanwhile, the naked polymers without LLL conjugation failed to display any Cas9 inhibition (Figure S2).

Furthermore, titration of PGA200-LLL resulted in a clear dose-dependent pattern of Cas9 inhibition, with a minimal PGA200-LLL/Cas9 molar ratio of 10:1 to completely block Cas9 activity. When we decreased the PGA200-LLL/Cas9 molar ratio to 5:1, Cas9 inhibition was partially abolished, for we detected traces of digested DNA in the agarose gel (Figure 4B, left, red arrow). Similarly, we titrated the PLD200-LLL/Cas9 molar ratio from 1200:1 down to 150:1, and DNA digestion data demonstrated that, in order to completely inhibit the nuclease activity of the Cas9 protein, the minimally required polymer/RNP molar ratio was approximately 800:1 (Figure 4B, right), indicating a much lower Cas9 inhibitory efficiency than the other two polymers.

- DNA digestion screening of various LLL-conjugated polymers (PMLA, PGA200, PLD200, PAA, and HA) revealed two more Cas9 inhibitors.
- The dose-dependence of Cas9 inhibition by PGA200-LLL and PLD200-LLL: Besides PMLA-LLL, the other two polymers exhibited varying efficiency in the inhibition of Cas9 nuclease activity with a ranking order of PMLA-LLL > PGA200-LLL > PLD200-LLL.
- The length of nano-polymer affects the Cas9 inhibitory efficiency. We evaluated the impact of the size/length of the nano-polymer on Cas9 inhibition using PGA as an example. The shortest PGA20-LLL cannot completely block Cas9-mediated DNA cleavage even with a polymer/RNP molar ratio of 50:1 (left panel). When comparing LLL-conjugated PGA20 vs. PGA50 vs. PGA100 with the same concentration (polymer/RNP = 20:1), only the longest PGA100-LLL exhibited 100% Cas9 inhibition (right panel).

### 3.3. Effects of Polymer Length on Cas9 Inhibition

By screening five different LLL-conjugated nano-polymers, we identified three of them as Cas9 inhibitors with varying inhibitory efficiencies with a ranking order of PMLA-LLL > PGA200-LLL > PLD200-LLL. We further asked whether the length of the polymers has any impact on the Cas9 inhibitory efficiency. To answer this question, we first chose a short PGA20 conjugated with LLL (PGA20-LLL) and determined its Cas9 inhibition efficiency with various concentrations. The DNA cleavage results showed that even with a PGA20-LLL/Cas9 molar ratio of 50:1, PGA20-LLL was still unable to completely inhibit Cas9 function with traces of digested DNA (Figure 4C, left). In comparison, PGA200-LLL only required a molar ratio (polymer/Cas9) of 10:1 to reach complete Cas9 inhibition (Figure 4B, left). Lastly, we compared the PGA-LLL polymers with three different backbone lengths (PGA20, 50, and 100) in their ability to inhibit Cas9 function. All three polymers were incubated with Cas9 at a polymer/Cas9 molar ratio of 20:1, and we found that only the longest PGA100-LLL exhibited a complete inhibitory effect, while both PGA20-LLL and PGA50-LLL showed slight traces of digested DNA in the gel (Figure 4B, right), indicating that longer forms of polymers have a stronger Cas9 inhibition effect.

### 3.4. Transmission Electron (TEM) Imaging

**Figure 5:**
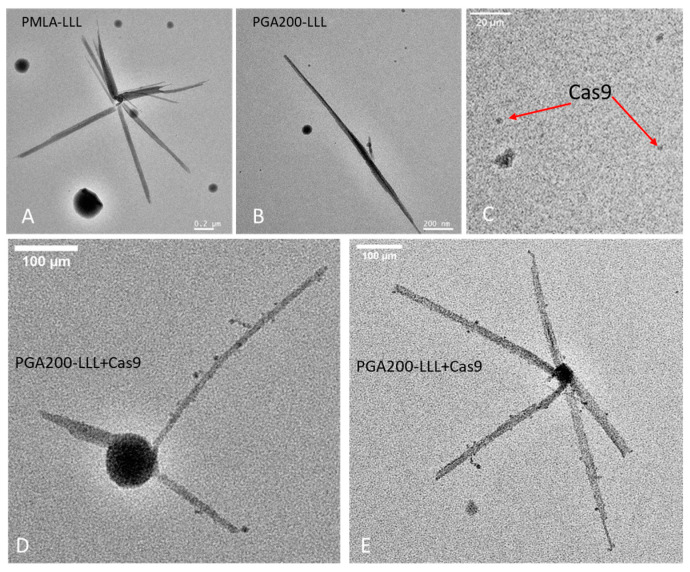
Representative transmission electron microscopy (TEM) images.

To understand the structural basis of Cas9-inhibitor polymers, we performed imaging by transmission electron microscopy using PMLA-LLL and PGA200-LLL. The TEM images demonstrated that both polymers have a linear conformation of an elongated, thread-like shape (Figure 5). PGA200-LLL exhibited a more straight and elongated structure under the TEM scan, while PMLA-LLL appeared as a bundle of fibers. Regardless, both polymers exhibited typical morphological features of linear structures. In contrast, the Cas9 protein, although with a molecular weight of >160 KD, appeared much smaller in TEM imaging and it displayed a blurred globular shape, reflecting its relatively more uniform electron densities than those of the nano-polymers as observed via TEM. When we combined the PGA200-LLL polymer and Cas9, the TEM images showed a close spatial relationship between Cas9 and the nano-polymer, with a series of compact and rounded spherical dots (Cas9) in close proximity to the fiber-shaped polymer surface, indicating a spatially close relationship between Cas9 and PGA200-LLL (Figure 5, lower panels).

### 3.5. Computational Prediction of Cas9-Polymer-LLL Interaction

**Figure 6:**
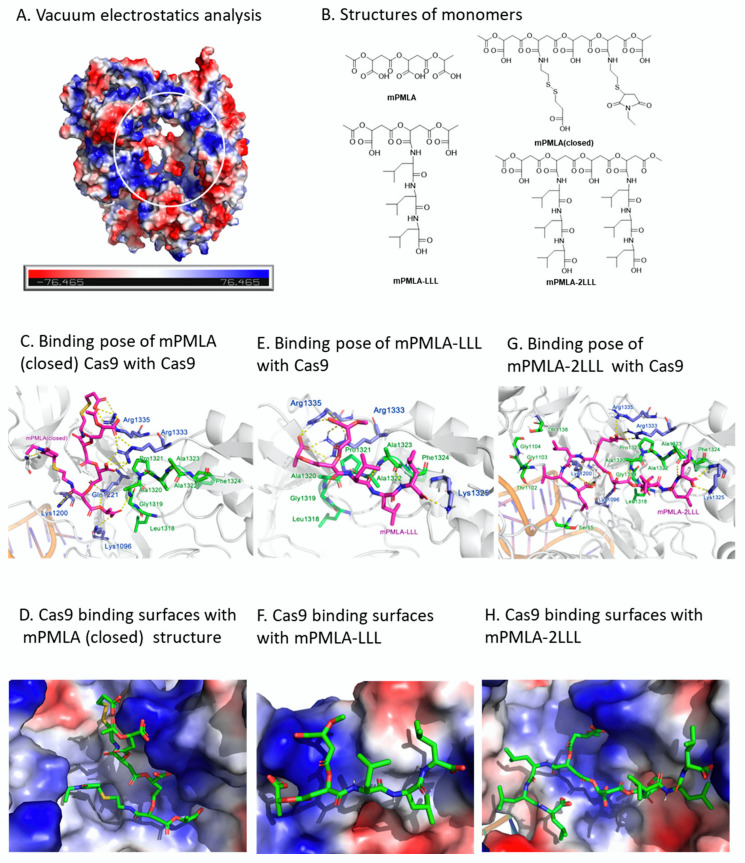
Molecular docking results of different monomers on Cas9.

The significant difference in Cas9 inhibition by PMLA and PMLA-LLL suggested that the polymer-conjugated LLL moiety could have unique interactions with Cas9 or the Cas9-DNA complex. The resolved structure of the SpCas9 protein in a complex with the protein inhibitor AcrIIA4 revealed that the dsDNA binding pocket is rich in positive amino acids [29]. These amino acids could form hydrogen bonds with AcrIIA4, or a ‘positive ring’ around the pocket to connect with dsDNA substrates (Figure 6A). Because the backbone of the PMLA polymer is rich in electronegative groups like ester and carboxyl, it is possible that PMLA may have a certain affinity with this pocket.

To explore the potential mechanism of Cas9 inhibition by PMLA-LLL, we performed molecular docking simulations to investigate the binding mode of PMLA-related monomers to Cas9, including a mono unit of PMLA (mPMLA), a capped mPMLA (closed), mPMLA conjugated with a LLL (mPMLA-LLL), or a double LLL (mPMLA-2LLL) (Figure 6B). After induced-fit docking and scoring, the top-scored pose of mPMLA-LLL revealed that the LLL moiety was close to a hydrophobic surface formed by Leu1318, Gly1319, Ala1320, Pro1321, Ala1322, Ala1323, and Phe1324 within the PAM-interacting domain (Figure S3A–C). Also, the connected PMLA backbone was close to a nearby hydrophilic site, including Arg1335 and Arg1333. Moreover, Lys1325 is another hydrophilic site next to the hydrophobic surface, which could form hydrogen bonds with the terminal carboxy group of LLL or other carboxy groups in the PMLA backbone (Figure S3A–C). Meanwhile, the docking results of the naked PMLA backbone predicted various interactions with the hydrophilic pocket, but only one interaction with the hydrophobic surface, suggesting the hydrophobic pocket may not be suitable to bind to the negatively charged PMLA backbone (Figure S3D–F).

Consistently, monomers with a cap moiety showed fewer effects on the polarity matching between the PAM-interacting domain and PMLA-LLL (Figure 6C,D). Furthermore, mPMLA-2xLLL showed that the adjacent LLL tends to be close to another hydrophobic site and did not disturb the interactions between Cas9 and mPMLA-LLL (Figure 6E–H). These results suggested that the hydrophobic surface of PAM could be a preferable site for the binding of the LLL moiety, and the inconsecutive hydrophilic sites within the pocket formed by spaced positive and neutral residues would be suitable for the interaction with PMLA-LLL but not naked PMLA, which could explain their difference in Cas9 inhibition.

A. Vacuum electrostatic analysis of SpCas9 protein: Blue, white, and red represents the positively, neutrally, and negatively charged surface. The white circle represents the ‘positive ring’ in the pocket.

B. The structures of monomers involved in the docking assay.

C, E, and G: The binding poses of the (C) mPMLA(closed) structure. (E) mPMLA-LLL and (G) mPMLA-2LLL. The monomers are represented in magenta, the hydrophobic residues are represented in green, and the residues with a positive charge are represented in blue.

D, F, and H: The Cas9 binding surfaces with the abovementioned polymers. The monomers are represented in green; blue, white, and red represent the positively, neutrally, and negatively charged surfaces.

## 4. Discussion

The repertoire of CRSIPR-Cas inhibitors has been rapidly expanding with new tools available to search for and identify these molecules. By origin, the Cas9 inhibitors can be divided into two groups: (1) naturally occurring Cas9 inhibitors, such as the Acr proteins; and (2) engineered Cas9 inhibitors. Recently, a machine-learning-based approach predicted over 2500 candidate anti-CRISPR (Acr) families in phage [30], indicating the existence of a gigantic number of Acr proteins in nature. More than 80 distinct Acr families have been identified to date that inhibit type I, II, III, V, and VI CRISPR–Cas systems [31], among which AcrIIA and AcIIC are Cas9-specific. Acr families have diverse modes of interaction with Cas proteins. As for the type-II Acr proteins, their inhibition mechanisms include the following: (1) inhibition of sgRNA binding (e.g., AcrIIA17 and AcrIIC2) [32,33]; (2) blocking target DNA entry by occupying the PAM binding site (e.g., AcrIIA2, AcrIIA4 and AcrIIC5) [22,29,34,35]; (3) interacting with the Cas9 catalytic domain (e.g., AcrIIC1 and AcrIIC3) [36,37,38] to inhibit its function; and (4) other unclear CRISPR inhibitory mechanisms, such as a dimerized AcrIIA1 with a nuclear acid binding affinity [39].

In addition to natural Acr proteins, researchers have engineered synthetic inhibitors to achieve more precise control over Cas9 activity. These synthetic inhibitors often aim to increase the specificity of genome editing or enable conditional activation or repression of Cas9. These engineered Cas9 inhibitors include both small molecules and peptide-based Cas9 inhibitors through rational design and screening [40,41]. To the best of our knowledge, our current work is the first report of nano-polymer Cas9 inhibitors identified to date. More interestingly, the linear polymer backbone conjugated with the common trileucine peptide appeared to be a common structural basis for this new class of Cas9 inhibitors to block the endonuclease function of Cas9. The LLL moiety has been previously used as an efficient mechanism for nanodrugs to escape endosomes and avoid degradation, and our current work has added a new layer of functional complexity to it in the potential regulation of Cas9 function.

All five different nano-polymers we screened for potential Cas9 inhibition are over 50,000 Daltons in molecular weight, and three of them exhibited varying efficiency in blocking Cas9 nuclease function. The largest polymer, PMLA-LLL, displayed the strongest Cas9 inhibition with a minimal polymer/RNP molar ratio of 3:1 to completely inhibit Cas9-mediated DNA cleavage, followed by PGA200-LLL and PLD200-LLL, sequentially. The HPLC spectra provide robust evidence of the dose-dependent binding between PMLA-LLL and Cas9. The reduction in the free Cas9 peak and the progressive increase in the PMLA-LLL/Cas9 complex peak support the high binding efficiency of PMLA-LLL. This behavior underscores its capability to encapsulate and stabilize the Cas9 protein effectively. Although PGA200-LLL and PLD200-LLL are quite close in their overall size (Table 3), the two polymers showed a substantial difference in their efficiency as Cas9 inhibitors (Figure 3), suggesting that the polymer structural differences are the major determinants of the Cas9 inhibitory efficiency. Interestingly, with the same backbone structure, there appears to be a requirement of length of the polymers to block Cas9 function, as the shorter forms of PGA-LLL showed a decreased efficiency in Cas9 inhibition.

This study also has several limitations. Firstly, although theoretical polymer–Cas9 binding models were generated computationally, the definitive structure of nano-polymer Cas9 inhibitor binding to the Cas9 protein still need to be established. Secondly, the stability of nano-polymer-LLL Cas9 inhibitors needs to be determined to better evaluate their potential in CRISPR/Cas9 functional regulation. Lastly, our data are limited to SpCas9 inhibition, and it is not clear whether this polymer–LLL structure can be a universal basis for the inhibition of Cas9 proteins of other origins, such as Neisseria meningitidis Cas9 (NmeCas9) [42], Staphylococcus aureus Cas9 (SaCas9) [43], and Campylobacter jejuni Cas9 (CjCas9) [44].

Many efforts have been made to increase the accuracy of Cas-mediated gene editing [45,46,47]. Our discovery of nano-polymers as Cas9 inhibitors raised an interesting possibility to enhance both CRISPR/Cas9 delivery and the precision of gene editing. First of all, these nano-polymers can be customized to target different cell types and disease models. This versatility means that a nano-polymer Cas9 inhibitor could be tailored to various therapeutic contexts, from cancer treatment to genetic disorders. For instance, PMLA-LLL-based nanodrugs have been explored in both brain tumor and Alzheimer’s disease settings from our previous work [10,30,48]. Secondly, the nano-polymers we identified with Cas9 inhibitory effects can potentially enhance the precision of CRISPR/Cas9-mediated gene editing by offering a temporal on/off switch over CRSIPR/Cas9 function. Nonetheless, our study has identified a new class of Cas9 inhibitors in nano-polymer form. Due to their biocompatibility and pH sensitivity, further efforts are warranted to develop them into dual functional CRISPR/Cas9 delivery carriers with functional regulation of Cas9 activity.

## 5. Patents

The findings of this work have been disclosed to and are currently under review by the Tech Transfer Office at the Cedars Sinai Medical Center for a patent filing.

## Figures and Tables

**Table 1:** Zeta potential measurement.

Product	Zeta Potential (mV) Buffer: H_2_O
PMLA	−5.6
PMLA + Cas9	−11.5
PMLA-LLL	−6.16
PMLA-LLL + Cas9	−22.1
PMLA-LLL + Cas9 + sgRNA	−24
Cas9	10.8
sgRNA	−4.1
Cas9 + sgRNA	−7.1
sgRNA + Cas9 + PMLA-LLL	−23.5
sgRNA + PMLA-LLL	−9.6
sgRNA + PMLA-LLL + Cas9	−19.7

**Table 2:** Polymer–Cas9 binding affinity determined by MST assays.

No.	Target	Ligand	K_D_ (nM)
1.	Cas9	sgRNA	1.24
2.	Cas9	PMLA-LLL	3.6
3.	Cas9	PMLA	36
4.	Cas9 + PMLA-LLL	sgRNA	528
5.	Cas9 + PMLA	sgRNA	23

PMLA-LLL is a bona fide Cas9 inhibitor.

**Table 3:** Summary of polymers in this study.

Polymer	Chemical Structure	Characteristic	Molecular Weight (MW)	MW after LLL Conjugation
PMLA	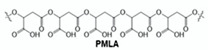	pH-sensitive	50,000 (116 per unit)	111,504 (258.7 per unit)
Poly-L-glutamic acid (PLE-200, or PGA-200)	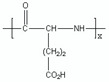	pH-sensitive	26,000 (129 per unit)	56,140 (280 per unit)
Poly-L-aspartic acid (PLD-200)	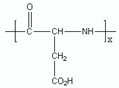	pH-sensitive	23,000 (115 per unit)	51,540 (257.7 per unit)
PAA (polyacrylic acid)	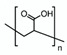	pH-sensitive	240,000, (25% is H_2_O) (72 per unit)	423,895 (127.25 per unit)
Hyaluronic acid (HA)	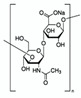	pH-sensitive	30,000 (403 per unit)	57,162 (768.3 per unit)

## Data Availability

All data are incorporated into the article and its online Supplementary Material.

## Supplementary Materials

The following supporting information can be downloaded at: https://www.mdpi.com/article/10.3390/polym17030417/s1.
