# Supplementary material for: Nano-Polymers as Cas9 Inhibitors
Source: Polymers (Basel). 2025 Feb 5;17(3):417. doi: 10.3390/polym17030417 (PMC11820846; doi:10.3390/polym17030417)
Supplement: Supplementary file 1 [file polymers-17-00417-s001.zip › polymers-3333323-supplementary.pptx]

## Slide 1
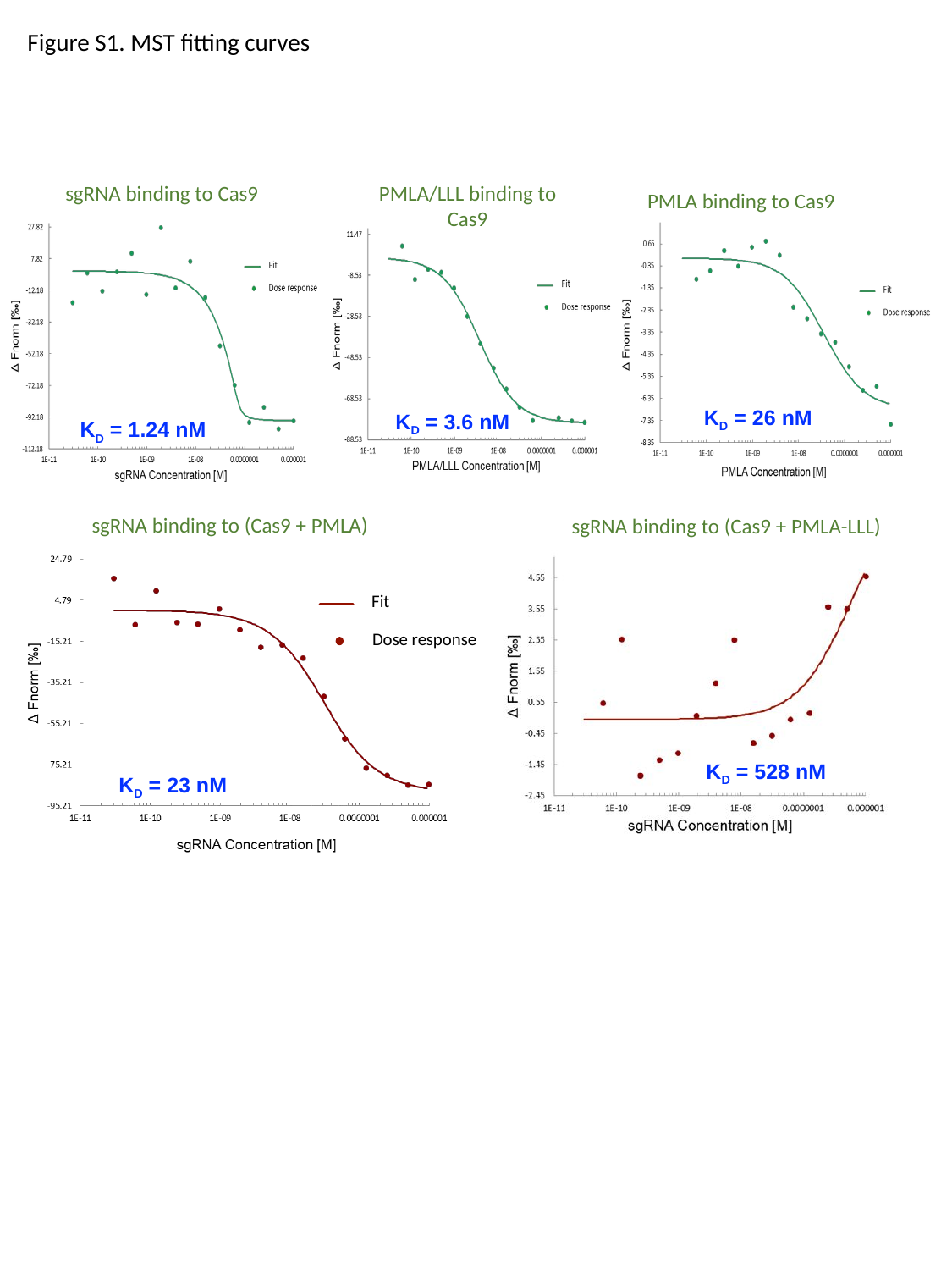

Figure S1. MST fitting curves
sgRNA binding to Cas9
PMLA/LLL binding to Cas9
KD = 3.6 nM
KD = 1.24 nM
PMLA binding to Cas9
KD = 26 nM
sgRNA binding to (Cas9 + PMLA)
sgRNA binding to (Cas9 + PMLA-LLL)
Fit
Dose response
KD = 528 nM
KD = 23 nM

## Slide 2
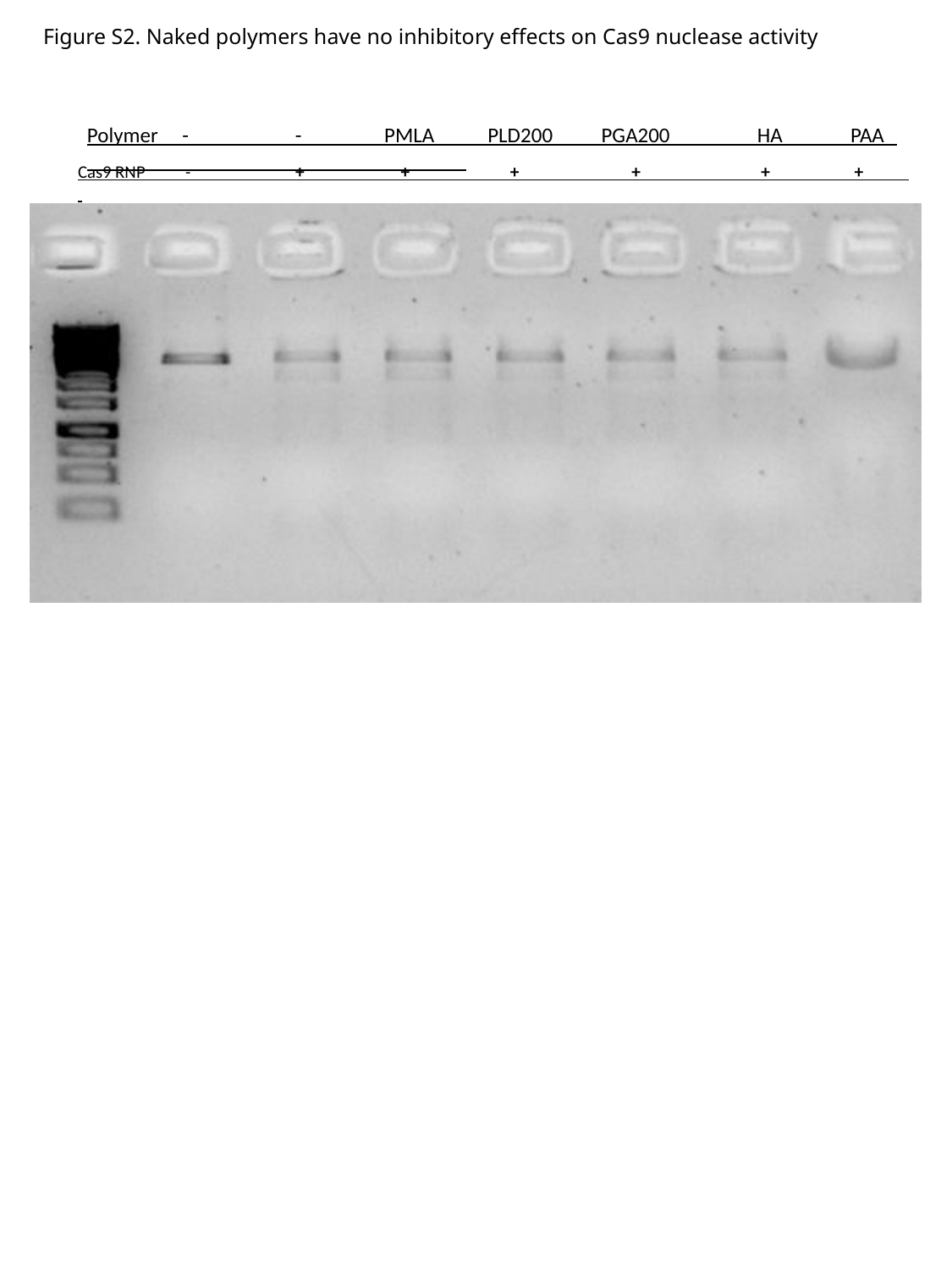

# Figure S2. Naked polymers have no inhibitory effects on Cas9 nuclease activity
Polymer - - PMLA PLD200 PGA200 HA PAA
Cas9 RNP - + + + + + +

## Slide 3
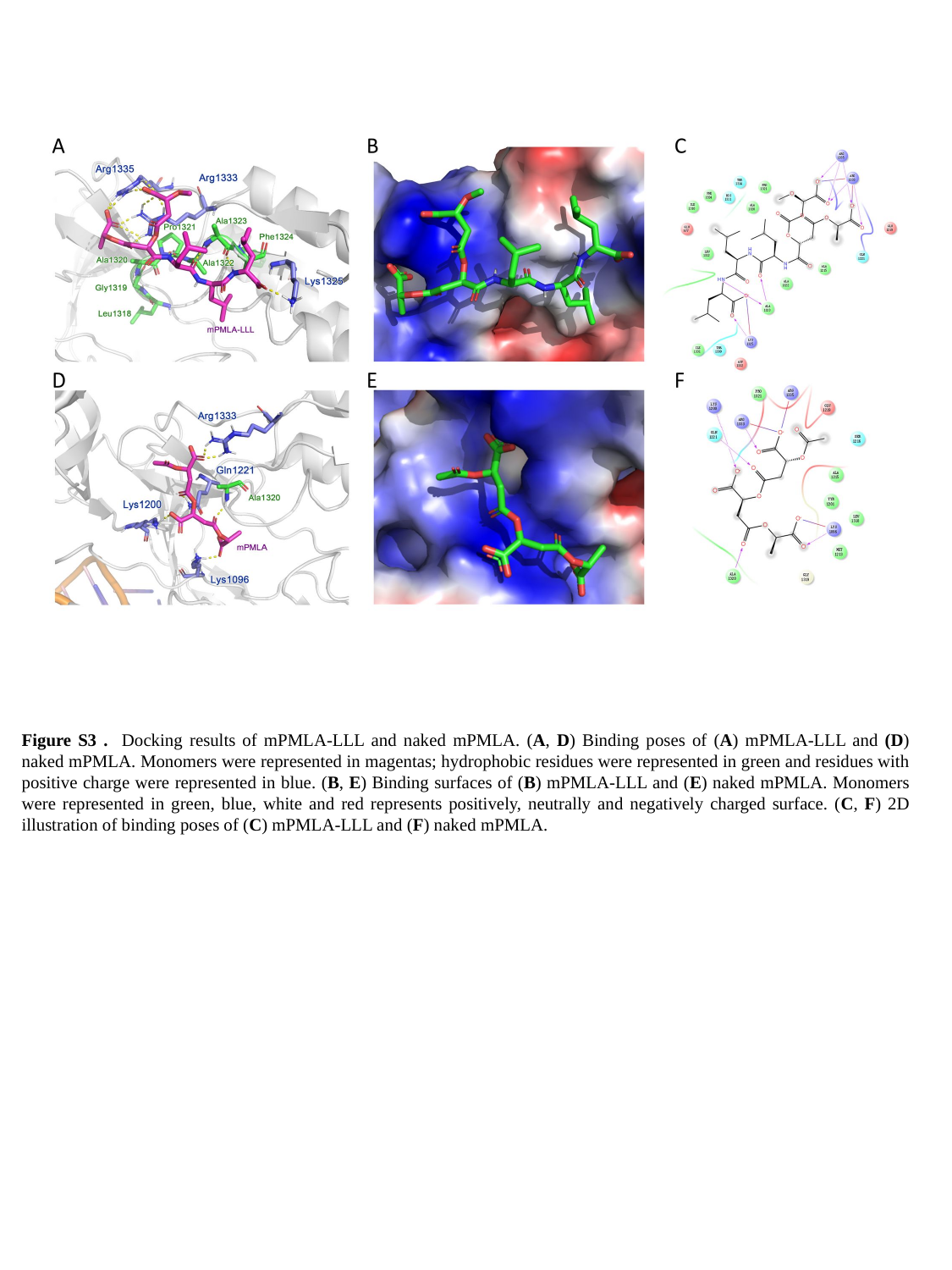

Figure S3 . Docking results of mPMLA-LLL and naked mPMLA. (A, D) Binding poses of (A) mPMLA-LLL and (D) naked mPMLA. Monomers were represented in magentas; hydrophobic residues were represented in green and residues with positive charge were represented in blue. (B, E) Binding surfaces of (B) mPMLA-LLL and (E) naked mPMLA. Monomers were represented in green, blue, white and red represents positively, neutrally and negatively charged surface. (C, F) 2D illustration of binding poses of (C) mPMLA-LLL and (F) naked mPMLA.
